# Molecular hydrogen treatment improves cognitive problems in Alzheimer's model

**DOI:** 10.1002/alz70855_100382

**Published:** 2025-12-23

**Authors:** Mariane Cristine Vicente, Arthur Dalmolin Dahmer, Kênia Cardoso Bícego, Luciane Helena Gargaglioni Batalhão

**Affiliations:** ^1^ São Paulo State University (UNESP), Jaboticabal, Brazil; ^2^ Paulista State University Júlio de Mesquita Filho, Faculty of Agricultural and Veterinary Sciences of Jaboticabal, Jaboticabal, São Paulo, Brazil

## Abstract

**Background:**

The literature to oxidative stress as a key contributor to neurodegeneration and neuroinflammation in Alzheimer's disease (AD). Consequently, antioxidant therapies can have a lasting impact for improving the quality of life of AD patients. By strengthening the antioxidant defenses, these therapies may prevent or minimize respiratory and cognitive symptoms characteristic of the disease. Thus, we hypothesize that molecular hydrogen is a potential candidate for the prevention and treatment of AD since it is a gas with antioxidant, anti‐apoptotic and anti‐inflammatory effects. Thus, the present study evaluated whether H_2_ treatment prevents and/or minimizes the molecular, cognitive and respiratory alterations caused by AD in experimental model for AD.

**Method:**

We employed a sporadic AD model induced in mice (*n* = 17_ by intracerebroventricular streptozotocin (STZ) injections at a dose of 3 mg/kg. The animals were subsequently exposed to 2% hydrogen (H_2_) for 5 (*n* = 5) and 7 days (*n* = 7). Learning and memory were evaluated by Barnes Maze. Ventilation and metabolism were measured via plethysmography under normocapnia and hypercapnia (7% CO_2_). In addition, we evaluated oxidative stress markers, including superoxide dismutase (SOD) and lipid peroxidation (LPO) levels.

**Result:**

Microinjection of STZ at a dose of 3 mg/kg increased ventilation in normocapnia, but not in hypercapnia. We did not observe changes in O_2_ consumption (VO_2_) and respiratory coefficient (V_E_/VO_2_) of STZ and vehicle animals. Likewise, we did no observe changes in body temperature (Tb) in normocapnia and hypercapnia between the groups, as well as no changes in the dosage of SOD and LPO. Our cognition results showed that 7 days exposure to H_2_ gas improved learning by 62% in time compared to STZ animals in room air.

**Conclusion:**

We conclude that the dose of 3 mg/kg promotes cognitive and respiratory changes present in AD, thus becoming a dose for a model of sporadic Alzheimer's in the intermediate and late window of the disease. In addition, treatment with H_2_ gas is effective in promoting improvements in the cognition of animals, making it a potential therapeutic candidate for AD.

